# Synthesis and cytotoxic properties of novel (*E*)-3-benzylidene-7-methoxychroman-4-one derivatives

**DOI:** 10.1186/2008-2231-21-31

**Published:** 2013-04-12

**Authors:** Saeedeh Noushini, Eskandar Alipour, Saeed Emami, Maliheh Safavi, Sussan Kabudanian Ardestani, Ahmad Reza Gohari, Abbas Shafiee, Alireza Foroumadi

**Affiliations:** 1 Department of Chemistry, Islamic Azad University, Tehran-North Branch, Zafar St, Tehran, Iran; 2 Department of Medicinal Chemistry and Pharmaceutical Sciences Research Center, Faculty of Pharmacy, Mazandaran University of Medical Sciences, Sari, Iran; 3 Institute of Biochemistry and Biophysics, University of Tehran, Tehran, Iran; 4 Medicinal Plants Research Center, Tehran University of Medical Sciences, Tehran, Iran; 5 Department of Medicinal Chemistry, Faculty of Pharmacy and Pharmaceutical Sciences Research Center, Tehran University of Medical Sciences, Tehran, Iran

**Keywords:** Synthesis, Chalcones, Cytotoxic activity, Cancer, Chroman-4-one

## Abstract

**Background and the purpose of the study:**

There has been increscent interest in the field of cancer chemotherapy by discovery and development of novel agents with high efficacy, low toxicity, and minimum side effects. In order to find new anticancer agents, we replaced the pyrazolone part of well-known cytotoxic agent SJ-172550 with 7-methoxychroman-4-one. Thus, a novel series of 3-benzylidene-4-chromanones were synthesized and tested in vitro against human cancer cell lines.

**Methods:**

The title compounds were prepared by condensation of 7-methoxychroman-4-one with suitable aldehydes in appropriate alcohol in the presence of gaseous HCl. The antiproliferative activity of target compounds were evaluated against MDA-MB-231 (breast cancer), KB (nasopharyngeal epidermoid carcinoma) and SK-N-MC (human neuroblastoma) cell lines using MTT assay.

**Results:**

Although the direct analog of SJ-172550 (compound **5d**) did not show any cytotoxic activity against tested cell lines, but 2-(2-chloro-6-methoxyphenoxy)acetic acid methyl ester analog **5c** showed some activity against MDA-MB-231 and SK-N-MC cells. Further modification of compound **5c** resulted in the 3-chloro-4,5-dimethoxybenzylidene derivative **5b** which demonstrated better cytotoxic profile against all tested cell lines (IC_50_ values = 7.56–25.04 μg/ml).

**Conclusion:**

The results demonstrated that the cytotoxic activity of compound **5b** against MDA-MB-231 and SK-N-MC cells is more than etoposide. Therefore, compound **5b** prototype could be considered as novel cytotoxic agent for further developing new anticancer chemotherapeutics.

## Introduction

Cancer has been known as one of the most impressive clinical problems in both developing and developed countries. In spite of improved diagnostic techniques and advances in prevention and chemotherapeutic management of cancer, the disease still afflicts millions of peoples in the world [1]. Cancer cells are defined by uncontrolled replications associated with self-sufficiency in growth signals, hyposensitivity to anti-growth signals, ongoing angiogenesis, metastasis, and evasion of apoptosis [2]. Anti-cancer agents cannot recognize cancer cells from normal cells, as a matter of fact, these agents usually act on metabolically active or rapidly proliferating cells [3]. Thus, there has been increscent interest in the field of cancer chemotherapy by discovery and development of novel agents with high efficacy, low toxicity, and minimum side effects.

During recent years, several researchers developed different chalcone-like compounds with anticancer activity through the introduction of heterocyclic scaffolds [4,5]. The chemical structure of chalcone is characterized by two aromatic rings connected by a three carbon, α,β-unsaturated carbonyl system (1,3-diphenyl-2-propen-1-one) [6-8]. The highly significant advantage of chalcone derivatives as cytotoxic agents is the low propensity to interact with DNA; which omits the risk of mutagenesity as the common side effect of current chemotherapeutic agents [9].

**Figure 1:**
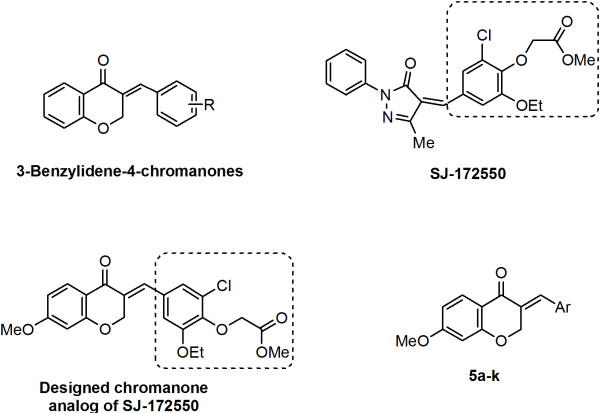
Structure of 3-benzylidene-4-chromanones as rigid analogs of chalcones exhibiting cytotoxic activity, structure of SJ-172550 as a lead compound that showed cytotoxic effects against tumour cell lines and designed compounds as cytotoxic agents.

Previously, Perjési et al. have reported cytotoxicity of 3-benzylidene-4-chromanones as rigid analogs of chalcones (Figure 1) [10]. Recently, high-throughput screening of drug libraries results in the identification of SJ-172550 that exhibited p53-dependent cytotoxic activity against cancer cell lines [11]. Structurally, SJ-172550 is characterized by having α,β-unsaturated carbonyl system attached to the 2-(2-chloro-6-ethoxyphenoxy)acetic acid methyl ester. Accordingly, in continuation of our research program to find novel anti-cancer agents [12-16] and considering the diverse biological activities of rigid chalcones [17], we have synthesized a series of 3-benzylidene-4-chromanones bearing 2-(2-chloro-6-alkoxyphenoxy) acetic acid esters. The related analogs of 3-benzylidene-4-chromanones were also prepared for more studying of structure-activity relationships (Figure 1).

## Material and methods

### Chemistry

**Scheme 1:**
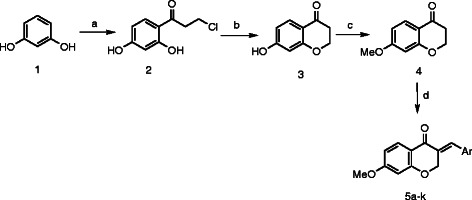
**General synthetic route to 3-benzylidene-4-chromanones 5a–k.***Reagents and conditions*: **(a)** 3-chloropropionic acid, CF_3_SO_3_H; **(b)** 2.0 M NaOH; **(c)** methyl iodide, K_2_CO_3_, DMF; **(d)** appropriate aldehyde, HCl (gas), ROH.

**Scheme 2:**
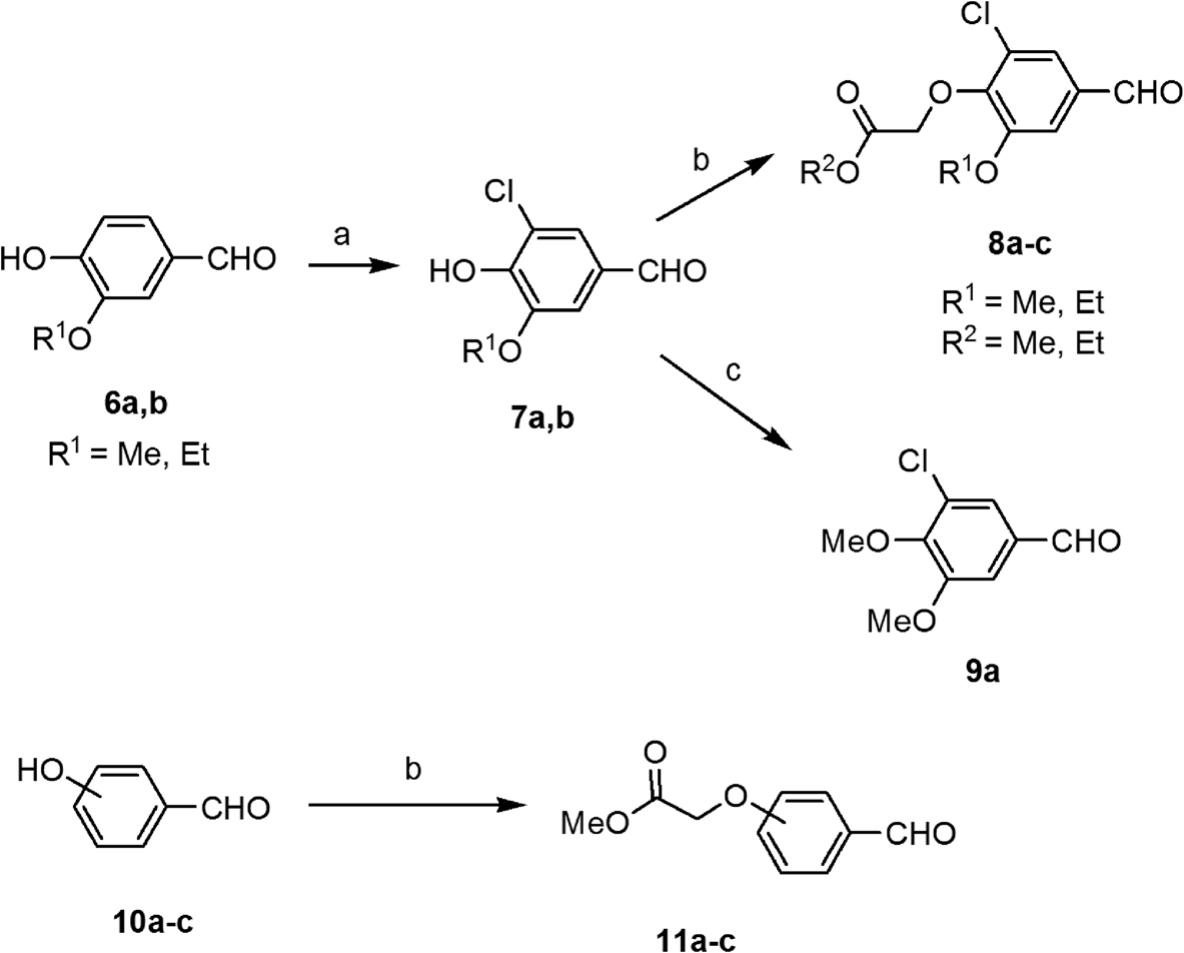
**Synthesis of aldehyde intermediates 7–9 and 11.***Reagents and conditions*: **(a)** Cl_2_ , CH_3_COOH; **(b)** R^2^OCOCH_2_Br, K_2_CO_3_, CH_3_COCH_2_CH_3_; **(c)** methyl iodide, K_2_CO_3_, DMF.

All chemical reagents and solvents were provided from Merck AG (Darmstadt, Germany). The general procedures for the synthesis of 3-benzylidene-4-chromanones **5a–k**, and aldehyde intermediates (compounds **7–9** and **11**) are illustrated in Schemes 1 and 2, respectively. 7-Methoxychroman-4-one (**4**) was prepared as literature method [18,19]. Melting points of compounds were determined using Kofler hot stage apparatus and are uncorrected. The IR spectra were recorded on a Shimadzu 470 spectrometer by using potassium bromide disks. The NMR spectra were obtained using a Bruker 400 MHz spectrometer (Bruker Bioscience, Billerica, MA, USA). Tetramethylsilane (TMS) was used as internal standard and chemical shifts (δ) are reported in ppm. Mass spectra were recorded on a Finnigan TSQ 70 spectrometer at 70 eV. Elemental analyses were carried out by using a HERAEUS CHN-O rapid elemental analyzer (Heraeus GmbH, Hanau, Germany) for C, H and N and the results are within ± 0.4% of the theoretical values.

#### Synthesis of 3-chloro-4-hydroxy-5-methoxybenzaldehyde (**7a**)

To a solution of vanillin (**6a**, 2.5 g, 16.4 mmol) in glacial acetic acid (15 ml) was slowly introduced a stream of chlorine gas over 30 min. White solid product was filtrated, washed with *n*-hexane (50 ml) to give compound **7a** (1.9 g). The acetic acid filtrate was again treated with chlorine gas flow as above for 30 min to give 0.7 g of compound **7a**[20]. A total of 2.6 g (85% yield) of white solid **7a** was obtained and used in next step without purification. ^1^H NMR (CDCl_3_, 400 MHz) δ: 9.7 (s, 1H, CHO), 7.5 (d, *J* = 1.6 Hz, 1H, aromatic), 7.3 (d, *J* = 1.6 Hz, 1H, aromatic), 3.9 (s, 3H, OCH_3_).

#### Synthesis of 3-chloro-5-ethoxy-4-hydroxybenzaldehyde (**7b**)

3-Ethoxy-4-hydroxybenzaldehyde (**6b**, 4 g, 5 mmol) was dissolved in a solution of glacial acetic acid (30 ml) and chloroform (10 ml) at 0°C and a stream of chlorine gas was slowly introduced over 15 min. Then, the solvents was evaporated and the residue was purified by silica gel column, eluting with a mixture of ethyl acetate/ petroleum ether (40:60) to give compound **7b** in 65% yield. ^1^H NMR (CDCl_3_, 400 MHz) δ: 10.3 (s, 1H, CHO), 7.5 (br s, 1H, aromatic), 6.9 (br s, 1H, aromatic), 5.7 (s, 1H, OH), 4.2 (q, *J* = 7.2 Hz, 2H, CH_2_), 1.5 (t, *J* = 7.2 Hz, 3H, CH_3_).

#### Synthesis of methyl 2-(2-chloro-4-formyl-6-methoxyphenoxy)acetate (**8a)**

A mixture of 5-chlorovanillin (**7a**, 2 g, 10.7 mmol) and K_2_CO_3_ (1.5 g, 10.8 mmol) in ethyl methyl ketone (40 ml) was stirred under reflux. After 10 min, methyl bromoacetate (1.64 g, 10.8 mmol) was added, and the mixture was allowed to stir under reflux for another 4 h. After the reaction was completed, ethyl methyl ketone was removed, and the residue was extracted with EtOAc (3 × 10 ml). The organic layer was dried over Na_2_SO_4_ and evaporated to give compound **8a** in 88.8% yield. ^1^H NMR (CDCl_3_, 400 MHz) δ: 9.8 (s, 1H, CHO), 7.4 (d, *J* = 1.6 Hz, 1H, aromatic), 7.3 (d, *J* = 1.6 Hz, 1H, aromatic), 4.8 (s, 2H, CH_2_), 3.9 (s, 3H, OCH_3_), 3.8 (s, 3H, OCH_3_).

#### Synthesis of methyl 2-(2-chloro-6-ethoxy-4-formylphenoxy)acetate (**8b)**

To a solution of compound **7b** (200 mg, 1 mmol) in ethyl methyl ketone (4 ml), was added potassium carbonate (147 mg, 1 mmol) and methyl bromoacetate (153 mg, 1 mmol) successively. The mixture was stirred under reflux for 2 h, cooled to room temperature and the solvent was removed under reduced pressure. The residue was added to 5 ml of water and extracted three times with ethyl acetate (5 ml). The combined extracts was dried (Na_2_SO_4_), and concentrated to give **8b** as a white solid in 91% yield. ^1^H NMR (CDCl_3_, 400 MHz) δ: 10.3 (s, 1H, CHO), 7.3 (s, 1H, aromatic), 6.8 (s, 1H, aromatic), 4.7 (s, 2H, CH_2_), 4.2 (q, *J* = 7.2 Hz, 2H, CH_2_), 3.8 (s, 3H, OCH_3_), 1.5 (t, *J* = 7.2 Hz, 3H, CH_3_).

#### Synthesis of ethyl 2-(2-chloro-6-ethoxy-4-formylphenoxy)acetate (**8c**)

To a solution of compound **7b** (500 mg, 2.5 mmol) in ethyl methyl ketone (10 ml), was added potassium carbonate (370 mg, 2.5 mmol) and ethyl bromoacetate (2.5 mmol) successively. The mixture was stirred under reflux for 3 h and then the solvent was removed under reduced pressure. The residue was added to 10 ml of water and extracted three times with ethyl acetate (10 ml). The combined extracts was dried (Na_2_SO_4)_ and concentrated to give **8c** as a white solid in 88% yield. ^1^H NMR (CDCl_3_, 400 MHz) δ: 10.2 (s, 1H, CHO), 7.3 (s, 1H, aromatic), 6.9 (s, 1H, aromatic), 4.7 (s, 2H, CH_2_), 4.25 (q, *J* = 7.2 Hz, 2H, CH_2_), 1.5 (t, *J* = 7.2 Hz, 3H, CH_3_), 1.3 (t, *J* = 7.2 Hz, 3H, CH_3_).

#### Synthesis of 3-chloro-4,5-dimethoxybenzaldehyde (**9a**)

To a solution of 5-chlorovanillin (**7a**, 5 g, 26.8 mmol) in DMF (40 ml) was added potassium carbonate (3.7 g, 26.8 mmol) and iodomethane (4.56 g, 32.16 mmol) successively. The mixture was stirred at 80°C for 3 h, cooled to room temperature and poured to water (100 ml). The precipitated white solid was filtrated and washed with water to give 5.1 g of compound **9a** in 94.8 yield. ^1^H NMR (CDCl_3_, 400 MHz) δ: 9.85 (s, 1H, CHO), 7.5 (s, 1H, aromatic), 7.35 (s, 1H, aromatic), 3.97 (s, 3H, OCH_3_), 3.94 (s, 3H, OCH_3_).

#### General procedure for the preparation of compounds **11a-c**

A mixture of hydroxybenzaldehyde **10a-c** (5 g, 40.95 mmol) and K_2_CO_3_ (6 g, 40.95 mmol) in ethyl methyl ketone (100 ml) was stirred under reflux. After 1 h, methyl bromoacetate was added, and the mixture was allowed to stir under reflux for another 3 h. After the reaction was completed, ethyl methyl ketone was removed, and the residue was extracted with EtOAc (3 × 20 ml). The organic layer was dried (Na_2_SO_4_) and evaporated to give methyl (formylphenoxy)acetate **11a-c**[21].

#### Methyl (2-formylphenoxy)acetate (**11a**)

This compound was obtained using general procedure as a pale yellow oil without further purification in 85% yield. IR (KBr, cm^-1^): 1767 (C = O, ester), 1690 (C = O, aldehyde); ^1^H NMR (CDCl_3_, 400 MHz) δ: 10.56 (s, 1H, CHO), 7.87 (dd, *J* = 5.6 and 2 Hz, 1H, H_6_-phenyl), 7.54 (t, *J* = 7.6 Hz, 1H, H_4_-phenyl), 7.09 (t, *J* = 7.6 Hz, 1H, H_5_-phenyl), 6.86 (t, *J* = 8.8 Hz, 1H, H_3_-phenyl), 4.7 (s, 2H, CH_2_), 3.8 (s, 3H, OCH_3_).

#### Methyl (3-formylphenoxy)acetate (**11b**)

This compound was obtained using general procedure as a pale yellow oil without further purification in 63% yield. IR (KBr, cm^-1^): 1761 (C = O, ester), 1682 (C = O, aldehyde); ^1^H NMR (CDCl_3_, 400 MHz) δ: 10 (s, 1H, CHO), 7.51 (m, 2H, H_5_, H_6_-phenyl), 7.36 (s, 1H, H_2_-phenyl), 7.25 (br s, 1H, H_4_-phenyl), 4.71 (s, 2H, CH_2_), 3.92 (s, 3H, OCH_3_).

#### Methyl (4-formylphenoxy)acetate (**11c**)

This compound was obtained using general procedure as a pale yellow oil without further purification in 93% yield. IR (KBr, cm^-1^): 1742 (C = O, ester), 1695 (C = O, aldehyde); ^1^H NMR (CDCl_3_, 400 MHz) δ: 9.9 (s, 1H, CHO), 7.9 (d, *J* = 8 Hz, 2H, aromatic), 7.0 (d, *J* = 8 Hz, 2H, aromatic), 4.73 (s, 2H, CH_2_), 3.82 (s, 3H, CH_3_O).

#### Synthesis of (E)-3-(3-chloro-4-hydroxy-5-methoxybenzylidene)-7-methoxychroman-4-one (**5a**)

A solution of 7-methoxychroman-4-one (**4**, 100 mg, 0.56 mmol), 5-chlorovanillin (**7a**, 105 mg, 0.56 mmol) in EtOH (2 ml) was stirred at room temperature for 5 min, while a stream of HCl gas was introduced. After 24 h at room temperature, the precipitation was filtrated, crystallized from EtOH to give **5a** as red solid in 52% yield. m.p. 171–173°C; IR (KBr, cm^-1^): 3428 (OH), 1655 (C = O); ^1^H NMR (DMSO-*d*_6_, 400 MHz) δ: 7.8 (d, *J* = 8.8 Hz, 1H, H_5_-chromanone), 7.6 (br s, 1H, CH-vinylic), 7.07 (d, *J* = 1.5 Hz, 1H, H_2_-phenyl), 7.03 (d, *J* = 1.6 Hz, 1H, H_6_-phenyl), 6.7 (dd, *J* = 6.4 and 2.4 Hz, 1H, H_6_-chromanone), 6.5 (d, *J* = 2.3 Hz, 1H, H_8_-chromanone), 5.4 (d, *J* = 1.6 Hz, 2H, H_2_-chromanone), 3.89 (s, 3H, OCH_3_), 3.83 (s, 3H, OCH_3_); Anal. Calcd for C_18_H_15_ClO_5_: C, 62.35; H, 4.36. Found: C, 62.04; H, 4.40.

#### Synthesis of (E)-3-(3-chloro-4,5-dimethoxybenzylidene)-7-methoxychroman-4-one (**5b**)

A solution of 7-methoxychroman-4-one (**4**, 500 mg, 2.8 mmol), 3-chloro-4,5-dimethoxybenzaldehyde (**9a**, 562 mg, 2.8 mmol) in EtOH (10 ml) was stirred at room temperature for 25 min, while a stream of HCl gas was introduced. After 24 h at room temperature, the precipitated solid was filtrated, crystallized from EtOH to afford compound **5b** as orange solid in 85% yield. m.p. 125–127°C; IR (KBr, cm^-1^): 1670 (C = O); ^1^H NMR (CDCl_3_, 400 MHz) δ: 7.96 (d, *J* = 8.8 Hz, 1H, H_5_-chromanone), 7.7 (br s, 1H, CH-vinylic), 6.8 (br s, 1H, H_2_-phenyl), 6.7 (br s, 1H, H_6_-phenyl), 6.6 (d, *J* = 8 Hz, 1H, H_6_-chromanone), 6.4 (br s, 1H, H_8_-chromanone), 5.3 (s, 2H, H_2_-chromanone), 3.92 (s, 3H, OCH_3_), 3.9 (s, 3H, OCH_3_), 3.85 (s, 3H, OCH_3_); Anal. Calcd for C_19_H_17_ClO_5_: C, 63.25; H, 4.75. Found: C, 62.88; H, 4.41.

#### Synthesis of (E)-methyl 2-(2-chloro-6-methoxy-4-((7-methoxy-4-oxo-2H-chromen-3(4H)-ylidene)methyl)phenoxy)acetate (**5c**)

A solution of 7-methoxychroman-4-one (**4**, 300 mg, 1.68 mmol), methyl 2-(2-chloro-4-formyl-6-methoxyphenoxy)acetate (**8a**, 435 mg, 1.68 mmol) in MeOH (6 ml) was stirred at room temperature for 20 min, while a stream of HCl gas was introduced. After 24 h at room temperature, the precipitation was filtrated, crystallized from MeOH to give compound **5c** as pink solid in 64% yield. m.p. 118–121°C; IR (KBr, cm^-1^): 1767 (C = O), 1750 (C = O); ^1^H NMR (CDCl_3_, 400 MHz) δ: 7.9 (d, *J* = 9.2 Hz, 1H, H_5_-chromanone), 7.7 (br s, 1H, CH-vinylic), 6.9 (s, 1H, H_3_-phenyl), 6.7 (s, 1H, H_5_-phenyl), 6.6 (d, *J* = 8.8 Hz, 1H, H_6_-chromanone), 6.4 (br s, 1H, H_8_-chromanone), 5.3 (s, 2H, OCH_2_CO), 4.7 (s, 2H, H_2_-chromanone), 3.87 (s, 3H, OCH_3_), 3.85 (s, 3H, OCH_3_), 3.83 (s, 3H, OCH_3_); Anal. Calcd for C_21_H_19_ClO_7_: C, 60.22; H, 4.57. Found: C, 60.36; H, 4.71.

#### Synthesis of (E)-methyl 2-(2-chloro-6-ethoxy-4-((7-methoxy-4-oxo-2H-chromen-3(4H)-ylidene)methyl)phenoxy)acetate (**5d**)

A solution of 7-methoxychroman-4-one (**4**, 200 mg, 1.12 mmol), methyl 2-(2-chloro-6-ethoxy-4-formylphenoxy)acetate (**8b**, 288 mg, 1.12 mmol) in MeOH (4 ml) was stirred at room temperature for 25 min, while a stream of HCl gas was introduced. After 24 h at room temperature, the precipitated solid was filtrated, crystallized from MeOH to give pure compound **5d** as a pink solid in 52% yield. m.p. 151–153°C; IR (KBr, cm^-1^): 1759 (C = O), 1737 (C = O); ^1^H NMR (CDCl_3_, 400 MHz) δ: 7.9 (d, *J* = 8.8 Hz, 1H, H_5_-chromanone), 7.8 (br s, 1H, CH-vinylic), 6.9 (s, 1H, H_3_-phenyl), 6.6 (br s, 2H, H_6_-chromanone and H_5_-phenyl), 6.4 (br s, 1H, H_8_-chromanone), 5.1 (s, 2H, CH_2_), 4.7 (s, 2H, H_2_-chromanone), 4.1 (m, 2H, CH_2_), 3.85 (s, 3H, OCH_3_), 3.82 (s, 3H, OCH_3_), 1.5 (t, *J* = 6.8 Hz, 3H, CH_3_); Anal. Calcd for C_22_H_21_ClO_7_: C, 61.05; H, 4.89. Found: C, 60.83; H, 5.03.

#### Synthesis of (E)-ethyl 2-(2-chloro-6-ethoxy-4-((7-methoxy-4-oxo-2H-chromen-3(4H)-ylidene)methyl)phenoxy)acetate (**5e**)

A solution of 7-methoxychroman-4-one (**4**, 50 mg, 0.28 mmol), ethyl 2-(2-chloro-6-ethoxy-4-formylphenoxy)acetate (**8c**, 76 mg, 0.28 mmol) in EtOH (2 ml) was stirred at room temperature for 15 min, while a stream of HCl gas was introduced. After 24 h at room temperature, the precipitation was filtrated, crystallized from EtOH to give compound **5e** as a white solid in 43% yield. m.p. 122–123°C; IR (KBr, cm^-1^): 1748 (C = O); ^1^H NMR (CDCl_3_, 400 MHz) δ: 7.9 (d, *J* = 8.0, 1H, H_5_-chromanone), 7.8 (br s, 1H, CH-vinylic), 6.9 (s, 1H, H_3_-phenyl), 6.6 (br s, 2H, H_6_-chromanone and H_5_-phenyl), 6.4 (s, 1H, H_8_-chromanone), 5.1 (s, 2H, H_2_-chromanone), 4.6 (s, 2H, OCH_2_CO), 4.2 (m, 2H, CH_2_), 4.1 (m, 2H, CH_2_), 3.85 (s, 3H, OCH_3_), 1.4 (m, 3H, CH_3_), 1.3 (s, 3H, CH_3_); Anal. Calcd for C_23_H_23_ClO_7_: C, 61.82; H, 5.19. Found: C, 62.01; H, 4.89.

#### Synthesis of (E)-methyl 2-(4-((7-methoxy-4-oxo-2H-chromen-3(4H)-ylidene)methyl)phenoxy)acetate (**5f**)

A solution of 7-methoxychroman-4-one (**4**, 100 mg, 0.56 mmol), methyl 2-(4-formylphenoxy)acetate (**11c**, 109 mg, 0.56 mmol) in anhydrous MeOH (2 ml) was stirred at room temperature for 25 min, while a stream of HCl gas was introduced. After 24 h at room temperature, the precipitation was filtrated, crystallized from methanol to afford pure compound **5f** as a red solid in 71% yield. m.p. 131–133°C; IR (KBr, cm^-1^): 1760 (C = O), 1667 (C = O); ^1^H NMR (CDCl_3_, 400 MHz) δ: 7.8 (d, *J* = 8.4 Hz, 1H, H_5_-chromanone), 7.6 (s, 1H, CH-vinylic), 7.41 (d, *J* = 8.8 Hz, 2H, H_2_- and H_6_-phenyl), 7.05 (d, *J* = 8.4 Hz, 2H, H_3_- and H_5_-phenyl), 6.7 (d, *J* = 8.2 Hz, 1H, H_6_-chromanone), 6.5 (s, 1H, H_8_-chromanone), 5.4 (s, 2H, OCH_2_CO), 4.8 (s, 2H, H_2_-chromanone), 3.8 (s, 3H, OCH_3_), 3.7 (s, 3H, OCH_3_); MS, m/z: 354, 295, 281, 265, 253, 151, 131, 115, 77, 63, 45. Anal. Calcd for C_20_H_18_O_6_: C, 67.79; H, 5.12; Found: C, 68.00; H, 4.98.

#### Synthesis of (E)-ethyl 2-(4-((7-methoxy-4-oxo-2H-chromen-3(4H)-ylidene)methyl)phenoxy)acetate (**5g**)

A solution of 7-methoxychroman-4-one (**4**, 100 mg, 0.56 mmol), methyl 2-(4-formylphenoxy)acetate (**11c**, 109 mg, 0.56 mmol) in anhydrous EtOH (2 ml) was stirred at room temperature for 20 min, while a stream of HCl gas was introduced. After 24 h at room temperature, the precipitation was filtrated, crystallized from ethanol to give compound **5 g** as a red solid in 71% yield. m.p. 133–135°C; IR (KBr, cm^-1^): 1760 (C = O), 1659 (C = O); ^1^H NMR (DMSO-*d*_6_, 400 MHz) δ: 7.8 (d, *J* = 8.4 Hz, 1H, H_5_-chromanone), 7.6 (s, 1H, CH-vinylic), 7.41 (d, *J* = 8.8 Hz, 2H, H_2_- and H_6_-phenyl), 7.05 (d, *J* = 8.4 Hz, 2H, H_3_- and H_5_-phenyl), 6.7 (d, *J* = 8.2 Hz, 1H, H_6_-chromanone), 6.5 (s, 1H, H_8_-chromanone), 5.4 (s, 2H, OCH_2_CO), 4.8 (s, 2H, H_2_-chromanone), 3.8 (s, 3H, OCH_3_), 3.7 (s, 3H, OCH_3_); MS, m/z: 368, 281, 253, 164, 151, 131, 115, 77. Anal. Calcd for C_21_H_20_O_6_: C, 68.47; H, 5.47. Found: C, 68.27; H, 5.51.

#### Synthesis of (E)-propyl 2-(4-((7-methoxy-4-oxo-2H-chromen-3(4H)-ylidene)methyl)phenoxy)acetate (**5h**)

A solution of 7-methoxychroman-4-one (**4**, 300 mg, 1.68 mmol), methyl 2-(4-formylphenoxy)acetate (**11c**, 327 mg, 1.68 mmol) in *n*-PrOH (6 ml) was stirred at room temperature for 15 min, while a stream of HCl gas was introduced. After 24 h at room temperature, the precipitation was filtrated, crystallized from PrOH to give compound **5 h** as a pink solid in 47% yield. m.p. 102–105°C; IR (KBr, cm^-1^): 1758 (C = O), 1660 (C = O); ^1^H NMR (DMSO-*d*_6_, 400 MHz) δ: 7.8 (d, *J* = 8.4 Hz, 1H, H_5_-chromanone), 7.6 (s, 1H, CH-vinylic), 7.4 (d, *J* = 8.0 Hz, 2H, H_2_- and H_6_-phenyl), 7.0 (d, *J* = 8.4 Hz, 2H, H_3_- and H_5_-phenyl), 6.7 (d, *J* = 8.0 Hz, 1H, H_6_-chromanone), 6.5 (s, 1H, H_8_-chromanone), 5.4 (s, 2H, OCH_2_CO), 4.89 (s, 2H, H_2_-chromanone), 4.09 (t, *J* = 6.4 Hz, 2H, OCH_2_), 3.8 (s, 3H,OCH_3_), 1.6 (m, 2H, CH_2_), 0.87 (t, *J* = 7.2 Hz, 3H, CH_3_); MS, m/z: 382, 354, 295, 281, 265, 151, 131, 115, 77, 69, 57, 43. Anal. Calcd for C_22_H_22_O_6_: C, 69.10; H, 5.80. Found: C, 68.90; H, 6.07.

#### Synthesis of (E)-butyl 2-(4-((7-methoxy-4-oxo-2H-chromen-3(4H)-ylidene)methyl)phenoxy)acetate (**5i**)

A solution of 7-methoxychroman-4-one (**4**, 100 mg, 0.56 mmol), methyl 2-(4-formylphenoxy)acetate (**11c**, 109 mg, 0.56 mmol) in *n*-BuOH (2 ml) was stirred at room temperature for 10 min, while a stream of HCl gas was introduced. After 24 h at room temperature, the precipitation was filtrated, crystallized from *n*-BuOH to afford compound **5i** as a pink solid in 36% yield. m.p. 85–87°C; IR (KBr, cm^-1^): 1766 (C = O), 1664 (C = O); ^1^H NMR (DMSO-*d*_6_, 400 MHz) δ: 7.8 (d, *J* = 8.8 Hz, 1H, H_5_-chromanone), 7.6 (br s, 1H, CH-vinylic), 7.4 (d, *J* = 8.0 Hz, 2H, H_2_- and H_6_-phenyl), 7.0 (d, *J* = 8.4 Hz, 2H, H_3_- and H_5_-phenyl), 6.7 (d, *J* = 7.6 Hz, 1H, H_6_-chromanone), 6.5 (br s, 1H, H_8_-chromanone), 5.4 (s, 2H, OCH_2_CO), 4.8 (s, 2H, H_2_-chromanone), 4.1 (t, *J* = 7.6 Hz, 2H, OCH_2_), 3.8 (s, 3H, OCH_3_), 1.5 (m, 2H, CH_2_), 1.3 (m, 2H, CH_2_), 0.8 (t, *J* = 7.6 Hz, 3H, CH_3_); MS, m/z: 396, 281, 265, 253, 167, 149, 131, 115, 107, 81, 57, 41. Anal. Calcd for C_23_H_24_O_6_: C, 69.68; H, 6.10. Found: C, 69.80; H, 6.37.

#### Synthesis of (E)-methyl 2-(2-((7-methoxy-4-oxo-2H-chromen-3(4H)-ylidene)methyl)phenoxy)acetate (**5j**)

A solution of 7-methoxychroman-4-one (**4**, 100 mg, 0.56 mmol), methyl 2-(2-formylphenoxy)acetate (**11a**, 109 mg, 0.56 mmol) in anhydrous MeOH (2 ml) was stirred at room temperature for 35 min, while a stream of HCl gas was introduced. After 24 h at room temperature, the precipitation was filtrated, crystallized from methanol to give compound **5j** as yellow viscous oil in 32% yield. IR (KBr, cm^-1^): 1755 (C = O), 1664 (C = O); ^1^H NMR (CDCl_3_, 400 MHz) δ: 7.98 (br s, 1H, H_5_-chromanone), 7.96 (s, 1H, CH-vinylic), 7.35 (d, *J* = 7.6 Hz, 1H, H_3_-phenyl), 7.07 (m, 2H, H_4_- and H_5_-phenyl), 6.81 (d, *J* = 8 Hz, 1H, H_6_-phenyl), 6.62 (d, *J* = 7.6 Hz, 1H, H_6_-chromanone), 6.4 (s, 1H, H_8_-chromanone), 5.22 (s, 2H, OCH_2_CO), 4.7 (s, 2H, H_2_-chromanone), 3.8 (s, 3H, OCH_3_), 3.79 (s, 3H, OCH_3_); MS, m/z: 354, 295, 281, 265, 151, 131, 77, 67, 57, 43. Anal. Calcd for C_20_H_18_O_6_: C, 67.79; H, 5.12. Found: C, 68.02; H, 4.98.

#### Synthesis of (E)-methyl 2-(3-((7-methoxy-4-oxo-2H-chromen-3(4H)-ylidene)methyl)phenoxy)acetate (**5k**)

A solution of 7-methoxychroman-4-one (**4**, 100 mg, 0.56 mmol), methyl 2-(3-formylphenoxy)acetate (**11b**, 109 mg, 0.56 mmol) in anhydrous MeOH (2 ml) was stirred at room temperature for 30 min, while a stream of HCl gas was introduced. After 24 h at room temperature, the precipitation was filtrated, crystallized from methanol to give compound **5 k** as a pink solid in 79% yield. m.p. 111–114°C; IR (KBr, cm^-1^): 1758 (C = O), 1670 (C = O); ^1^H NMR (DMSO-*d*_6_, 400 MHz) δ: 7.8 (d, *J* = 8.8 Hz, 1H, H_5_-chromanone), 7.7 (s, 1H, CH-vinylic), 7.4 (t, *J* = 6.8 Hz, 1H, H_6_-phenyl), 7.0 (m, 3H, H_2_ and H_4_- and H_5_-phenyl), 6.7 (dd, *J* = 6.8 and 2.0 Hz, 1H, H_6_-chromanone), 6.5 (d, *J* = 2.0 Hz, 1H, H_8_-chromanone), 5.4 (s, 2H, OCH_2_CO), 4.9 (s, 2H, H_2_-chromanone), 3.8 (s, 3H, OCH_3_), 3.7 (s, 3H, OCH_3_**)**; MS, m/z: 354, 281, 178, 167, 150, 122, 107, 79, 69, 57, 43. Anal. Calcd for C_20_H_18_O_6_: C, 67.79; H, 5.12. Found: C, 67.65; H, 5.33.

### Cytotoxicity assay

The in-vitro cytotoxic activity of each synthesized compounds **5a-k** was assessed using MTT colorimetric assay according to the literature method [22]. Each set of experiments was independently performed three times. For each compound, the concentration causing 50% cell growth inhibition (IC_50_) compared with the control was calculated from concentration-response curves by regression analysis.

## Results and discussion

### Chemistry

Reaction sequence employed for the synthesis of (*E*)-3-benzylidene-7-methoxychroman-4-one derivatives **5a-k** is shown in Scheme 1. The reaction of resorcinol **1** with 3-chloropropionic acid in the presence of trifluoromethane sulfonic acid furnished 2′,4′-dihydroxy-3-chloropropiophenone (**2**) which was cyclized using 2 M NaOH to give 7-hydroxy-4-chromanone (**3**). Compound **4** was obtained by reacting intermediate **3** with iodomethane in the presence of potassium carbonate in DMF. Condensation of 7-methoxychroman-4-one (**4**) with suitable aldehydes **7**–**9** and **11** in appropriate alcohol in the presence of gaseous HCl gave the target compounds **5a-k**.

The corresponding aldehydes **7**–**9** and **11** were prepared as shown in Scheme 2. Chlorination of 3-alkoxy-4-hydroxybenzaldehyde **6a,b** using acetic acid as a solvent gave 3-chloro-4-hydroxy-5-alkoxybenzaldehyde **7a,b** which was reacted with suitable alkyl bromoacetate, in the presence of potassium carbonate to give compounds **8a-c**. On the other hand, *O*-methylation of compound **7a** afforded dimethoxybenzaldehyde derivative **9a**. Methyl (formylphenoxy)acetate **11a-c** was synthesized by heating compounds **10a-c** with methyl bromoacetate and potassium carbonate in ethyl methyl ketone.

### In vitro cytotoxic activity

The cytotoxic activity of synthesized compounds **5a-k** was evaluated against three cell lines namely MDA-MB-231 (breast cancer), KB (nasopharyngeal epidermoid carcinoma) and SK-N-MC (human neuroblastoma) cells. The results of cytotoxic assay were mentioned as IC_50_ (μg/ml) of compounds in comparison with reference drug etoposide in Table 1.

**Table 1:** **Cytotoxic activity (IC**_**50**_**, μg/ml) of compounds 5a-k against different cell lines in comparison with etoposide**


**Compounds**	**Ar**	**MDA-MB-231**	**KB**	**SK-N-MC**
**5a**		19.70 ± 3.07	36.85 ± 2.97	12.60 ± 8.45
**5b**		7.56 ± 2.23	25.04 ± 10.60	9.64 ± 2.71
**5c**		20.03 ± 4.27	>100	58.04 ± 21.08
**5d**		>100	>100	>100
**5e**		>100	>100	>100
**5f**		16.47 ± 1.47	>100	>100
**5g**		16.32 ± 2.67	>100	>100
**5h**		18.87 ± 0.43	>100	>100
**5i**		7.10 ± 2.99	>100	>100
**5j**		14.23 ± 4.37	>100	>100
**5k**		10.53 ± 0.86	>100	>100
**Etoposide**		21.2 ± 2.12	18.93 ± 1.78	14.04 ± 1.05

In the case of MDA-MB-231 cell line, the IC_50_ values of all compounds were ≤20 μg/ml with the exception of compounds **5d** and **5e**. Furthermore, compounds **5b** and **5i** exhibited the highest cytotoxic activity against this cell line (IC_50_ < 10 μg/ml). Compound **5b** was also the most potent derivative against KB cell line with IC_50_ value of 25.04 μg/ml. Beside compound **5b**, compound **5a** exhibited good activity against KB cells, but remaining compounds **5c-k** showed no activity against this cell line (IC_50_ >100 μg/ml). Against SK-N-MC cells, compound **5b** followed by compounds **5a** and **5c** showed significant inhibitory activity with IC_50_ values of 9.64, 12.6 and 58.04 μg/ml, respectively.

Overall, it is clear that among the test compounds described in this study, the 3-chloro-4,5-dimethoxybenzylidene derivative **5b** demonstrated better cytotoxic profile against all tested cell lines (IC_50_ values = 7.56–25.04 μg/ml). Generally, the comparison of IC_50_ values of compound **5b** with those of etoposide demonstrated that the cytotoxic activity of compound **5b** against MDA-MB-231 and SK-N-MC cells is more than etoposide.

In this work, as part of an ongoing program to find new cytotoxic agents, we have focused our attention on modification of the 3-benzylidene-4-chromanones and introducing new functionality on the benzylidene moiety. Thus, we designed novel 3-benzylidene-4-chromanones that possessed a 2-(2-chloro-6-alkoxyphenoxy)acetic acid ester. These modifications were made on the basis of SJ-172550, a new cytotoxic agent possessing 2-(2-chloro-6-ethoxyphenoxy)acetic acid methyl ester attached to the pyrazolone ring. Surprisingly, compound **5d**, the chromanone analog of SJ-172550 showed no activity against tested cell lines. Also, the ethyl ester counterpart of **5d** (compound **5e**) was inactive against tumor cell lines. However, the 2-(2-chloro-6-methoxyphenoxy)acetic acid methyl ester analog **5c** was active against MDA-MB-231 and SK-N-MC cells. We have briefly investigated the SAR of compounds by simplification of the functionality on the benzylidene part of the basic molecule.

As can be deduced from the cytotoxic data of compounds **5f-k** which characterized by the lack of 2-chloro-6-alkoxy functionality, the cytotoxic activity against MDA-MB-231 can be served by the simple phenoxyacetic acid ester derivatives. However, the lack of 2-chloro-6-alkoxy functionality results in the lack of activity against KB and SK-N-MC cells. Among the compounds **5f-k**, butyl ester derivative **5i** showed the highest activity against MDA-MB-231 being 3-fold more potent than standard drug etoposide.

To determine the effect of acetic acid ester substitution in compound **5c**, we prepared both the 4-hydroxy derivative **5a** and 4-methoxy analog **5b**. When compared to **5c**, both compounds had similar or better in vitro activities against tested cell lines.

The cytotoxic activities of regio-isomeric compounds **5f, 5j** and **5 k** against MDA-MB-231cells revealed that changing the position of oxyesteric group has led to non-significant changes in activities. As seen from data, in poly-substituted compounds changing of methoxy group on phenyl ring to ethoxy group (for example **5d** versus **5c**) dramatically decreased the cytotoxic potency in MDA-MB-231 and SK-N-MC cells.

In summary, in the pursuit for finding new cytotoxic agents, we replaced the pyrazolone part of well-known cytotoxic agent SJ-172550 with 7-methoxychroman-4-one. Although the direct analog of SJ-172550 (compound **5d**) did not show any cytotoxic activity against tested cell lines, but 2-(2-chloro-6-methoxyphenoxy)acetic acid methyl ester analog **5c** showed some activity against MDA-MB-231 and SK-N-MC cells. Further modification of compound **5c** resulted in the 3-chloro-4,5-dimethoxybenzylidene derivative **5b** which demonstrated better cytotoxic profile against all tested cell lines (IC_50_ values = 7.56–25.04 μg/ml).

It is worthwhile to mention that, since we have originally designed the target compounds based on p53-dependent cytotoxic agent SJ-172550, it was better using the latter compound as standard drug in our cytotoxic assay. However, our primary cytotoxic experiments on the closest compound to SJ-172550 (compound **5d**) in a side-by-side comparison manner with etoposide revealed that compound **5d** had no activity against cancer cell lines. On the other hand, simplified compounds **5a** and **5b** with more dissimilarity respect to the SJ-172550 showed better profile of cytotoxicity. Based on these results, it seems that a different mechanism is responsible for potential cytotoxic activity of compound **5b** prototype.

We employed MTT cell viability assay as a standard and well-documented in vitro method for evaluation of the cytotoxic potential of designed compounds. Although these types of in vitro models are beneficial and promising as early screening tools for finding new lead compounds, but these models are associated with some limitations [23]. Thus, for efficacy and safety evaluation of lead compounds, conducting a method based on animal model is necessary in the next steps of study.

In conclusion, the results demonstrated that the cytotoxic activity of 3-(3-chloro-4,5-dimethoxybenzylidene)-7-methoxychroman-4-one (**5b**) against MDA-MB-231 and SK-N-MC cells is more than standard drug etoposide. Therefore, compound **5b** prototype bearing 3-chloro-4,5-dimethoxybenzylidene moiety could be considered as novel lead compound for further developing new anticancer chemotherapeutics. Although, compound **5b** showed promising activity in vitro, but to identify a promising anticancer drug candidate that has good pharmacokinetic and toxicological profiles, the in vivo ADME-Tox studies of compound **5b** prototype should be conducted.

## Competing interests

The authors declare that they have no competing interests.

## Authors’ contributions

SN: Synthesis of target compounds. EA: Supervision of the synthetic part. SE: Collaboration in design and identifying of the structures of target compounds, manuscript preparation. MS: Performed the cytotoxic tests. SKA: Supervision of the cytotoxic tests. ARG: Collaboration in identifying the structures of target compounds. AS: Collaboration in identifying the structures of target compounds. AF: Design of target compounds and supervision of the synthetic and pharmacological parts. All authors read and approved the final manuscript.
